# Identification of factors for a successful implementation of medication reviews in community pharmacies: Using Positive Deviance in pharmaceutical care

**DOI:** 10.1007/s11096-021-01315-1

**Published:** 2021-08-06

**Authors:** Isabell Waltering, Oliver Schwalbe, Georg Hempel

**Affiliations:** 1 Institute of Pharmaceutical and Medicinal Chemistry, Clinical Pharmacy, Westfaelische Wilhelms-University Muenster, Corrensstrasse 48, 48149 Muenster, Germany; 2 Department of Education and Training, Pharmacists’ Chamber of Westphalia-Lippe, Bismarckallee 25, 48151 Muenster, Germany

**Keywords:** Community pharmacy, Implementation, Medication review, Positive Deviance, Success factor

## Abstract

*Background* Pharmacists in community pharmacies worldwide successfully conduct an increasing number of medication reviews (MR). Since June 2012 MR are incorporated in the German ordinance on the operation of pharmacies as pharmaceutical service. In November 2014, a German guideline for MR was established. Different teaching programmes for MR were implemented since. Despite these favorable conditions, only few pharmacies conduct MR regularly. *Objective*: Identification of factors necessary for a successful implementation of MR in community pharmacies. *Setting*: Community pharmacies located in the area of the Pharmacists’  Chamber Westphalia-Lippe (Part of Northrhine-Westphalia, Germany). *Method*: Following a Positive-Deviance approach, telephone interviews were conducted in community pharmacies with pharmacy-owners, MR-trained employed pharmacists, and technicians. Data evaluation was performed using qualitative content analysis. *Main outcome results*: Successful strategies for implementing MR in community pharmacies. *Results*: Forty-four interviews were conducted and analysed. Thirty-three success factors were identified. Data analysis revealed two groups of success-factors important for implementation of MR; organisational (n = 25) and individual factors (n = 8). Relevant organisational success-factor were involvement of the entire team with active involvement of technicians, documentation of results in the pharmacy software and training in patient-identification and communication. Restructuring of workflows increased time-periods for MR. Important individual success-factors were: motivation and identification with the service, routine in execution to enhance self-esteem, and specialisation in pharmacotherapy of particular diseases. Pharmacy-owners play a pivotal role as motivators. Professional healthcare attitude, exhibited in daily routine, leads to increased acceptance by patients and practitioners and thus increases implementation-rates considerably. *Conclusion*: We were able to define strategies for successful implementation of MR in community pharmacies.

**Supplementary Information:**

The online version contains supplementary material available at 10.1007/s11096-021-01315-1.

## Impact of practice

- Positive Deviance is a unique approach in health services research to identify strategies internally generated rather than externally stipulated.
- The identified success factors close the gap between barriers and facilitators for the implementation of MR known from literature and procedures functioning already in real life.
- The different factors identified can be used by individual pharmacies to tailor measures for optimisation of MR provision.
- Involvement of the entire team and ongoing training of all team members in the different aspects of MR are the most important organisational factors. Individual motivation and support from pharmacy owners is of utmost importance to conduct MR successfully.

## Introduction

**Fig. 1:**
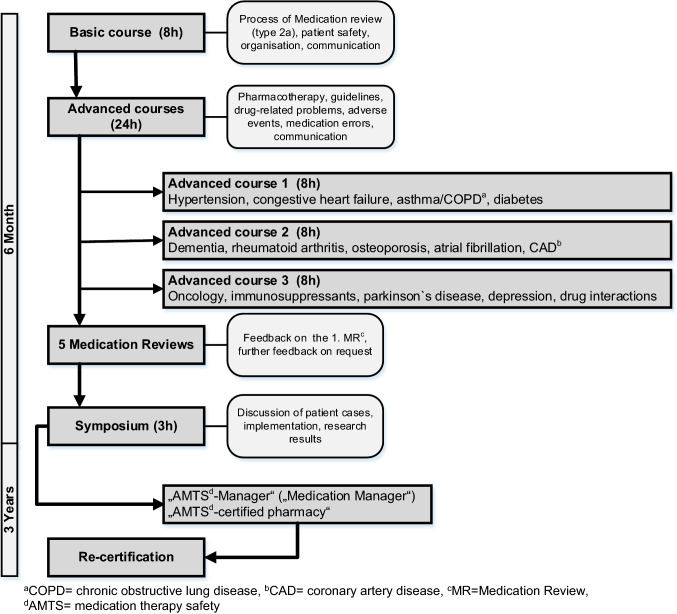
Course of the Apo-AMTS-programme

During the last decade, multi-morbidity, chronic illnesses and polymedication have increased, and are projected to increase further [1]. Therefore, numerous problems with medication therapy occur like non-adherence, over- and underprescribing, side-effects or drug-drug-interactions [2]. Medication reviews (MR) performed by community pharmacists can play a pivotal role in the management of these problems and are therefore already implemented as a cognitive service in several countries [3–7]. German pharmacies are facing a turning point with focusing on patient care by conducting MR rather than solely on dispensing. A first step was the incorporation of MR in the German ordinance on the operation of pharmacies as a pharmaceutical service in 2012 to establish a legal basis for this service [8]. A guideline for conducting MR was published in 2014 by the federal pharmacy chamber [9]. To support pharmacists/pre-registration-students in performing MR, a six month teaching programme called *Apo-AMTS-programme* (Fig. 1), has been implemented in Westphalia-Lippe; a region in Germany; since 2012 to impart competence on how to conduct intermediate MR Type 2a in line with the Pharmaceutical Care Network Europe (PCNE) definition [10, 11]. Even if *Apo-AMTS* is implemented in Westphalia-Lippe the programme is open for all pharmacists or pre-registration students in Germany.

In addition to training, several tools (e.g., documentation material, promotion material, hotline for pharmacotherapeutic questions) were developed to support implementation. An online survey executed in June 2016 revealed that only 53 (33%) of 163 certified pharmacies (*AMTS-qualified pharmacies*) conducted at least one MR per month, whereby 36% conducted no MR at all. Despite being trained and equipped with adequate material and having access to professional support, only a small number of pharmacies implemented this service for their patients on a regular basis. Facilitators and barriers to implement MR in community pharmacies like lack of time and staff, documentation of MR, hight demand of dispensing related activities were described in the literature previously, but they were derived from theory and formulated rather general and hence not applicable to German community pharmacies without modifications. It appears that little consideration has been given how facilitators like education and training for pharmacists, professional satisfaction and inclusion of technicians can best be used in practice to accelerate MR implementation and it seems important to identify facilitators at both individual and organisational level. We therefore used the *Positive Deviance* (PD) approach to assess practical measures already used successfully in a real life setting.

PD is an approach developed by Jerry and Monique Sternin,

- based on observations that in every community or profession a team or individual person exists
- that developed a more successful strategy or behavior compared to their peers, despite facing
- the same barriers and challenges and having no extra or the same resources or knowledge
- then their peers [12, 13].

Transfering PD to the disappointing situation with MR in trained pharmacies means that we included pharmacies conducting MR on a regular basis (*high performer*) to identify strategies and behaviors used in this group that have the potential to support the implementation of this service in all community pharmacies.

PD approaches were able to show impressive improvement in health-outcomes for complex problems globally (childhood nutrition, pregnancy outcomes, condom use) [14]. PD-studies are also frequently used in hospital settings e.g. to reduce the rates of nosocomial infections [15–18]. To the best of our knowledge, this is the first study applying PD to the community pharmacy sector in Europe.

### Aim of the study

Our aim was to identify success factors for implementation of medication reviews in German community pharmacies using the Positive Deviance approach.

### Ethic approval

According to the Fourth Act amending pharmaceutical legislation and other provisions, and the recommendations of the German Research Foundation for interview studies ethics approval are not necessary [19, 20]. The Clinical trial Regulation (Regulation EU No 536/2014) is actually non-applicable in Germany [21].

## Methods

### Study design

We used the PD approach to detect specific measures and actions used in community pharmacies that were able to conduct MR on a regular basis and integrate this service into their routine. PD is based on the assumption that a group of persons, here the pharmacy teams with higher rates of MR, act more successfully than their peers even though they use the same resources. Accordingly, same resources in our study are considered participation in a training-programme for pharmacists to conduct MR (*Apo-AMTS*) and access to all necessary material (e.g., documentation- and promotion-material) as well as professional support from the Pharmacists` Chamber Westphalia-Lippe. PD focusses on *how* procedures within the context of MR are transferred into practice instead of *what* is implemented. Therefore, this approach offers the unique opportunity to detect effective strategies compared to suggested measures from literature. To assure that these detected strategies are unique or at least exceptional, a control group (CG) was selected consisting of pharmacies not conducting MR despite being trained and equipped equally [14]. We performed the study from March 2017 until March 2019 in the area of the Pharmacists` Chamber of Westphalia-Lippe.

### Participants and recruitment

Recruitment of the different pharmacies followed the purposive sampling method [22]. All eligible pharmacies were certified *AMTS-pharmacies (see Fig. **1**)* (N = 354) and additionally enrolled in a collaboration project with a health insurance company (3A-project). Within this project, pharmacies received 80 € for every documented MR. *AMTS-pharmacies* enrolled in the *3A-project* with the highest count of documented MR from December 2016 until March 2017 were selected for the PD-group as *high-performers*; enrolled pharmacies with no MR during this time frame were selected as control-group (CG). Inclusion criteria for the pharmacies in both groups was the engagement of at least one *AMTS-manager* (trained pharmacist with successful completion of the *AMTS-programme*) to assure an equal baseline situation according to organisational structures. To assess regional effects, pharmacies were additionally stratified by city-size and their geographical position within Westphalia-Lippe [23]. An *AMTS-manager*, a technician and the pharmacy-owner/branch-manager from each pharmacy were invited for participation (professional groups). All participants received an information sheet including aim and structure of the study and a consent form for recording of the interviews. A sample size of six pharmacies for each group should be reached based on a recommendation of Rose et al. [24].

### Data collection

For data collection we conducted semi-structured interviews. For this purpose, interview-guides for each professional group were developed and pilot-tested. Questions for the interview-guides were derived from literature and personal experience of IW (expertise in conducting MR and training in development of questionnaires). For this pilot-test, twelve interviews were performed with two interviewees in each professional group within both the PD-group and CG. A modification of the interview-guides followed this test mainly by rephrasing to perceive more questions that are open-ended. IW conducted all interviews. The interview-guides are available in Supplement 1. Audio recording was performed using Audacity® (Audacity Incorporation, Version 2.2.2). Interviews were de-identified (identification numbers for participants/pharmacies) and a verbatim transcript was created (f4transkript v6/f5transkript v3, Dresing & Pehl GmbH, Marburg). Additionally, age, gender and years of professional experience were documented for each participant and each participant provided written consent to recording and analysis of the data.

### Data analysis

A qualitative content analysis based on Mayring et al. was performed [25]. A coding-system was developed, using a deductive-inductive approach. Therefore, based on the interview-guides, a first set of main codes was deviated and amended by sub-codes retrieved from the interview-transcripts. Two raters tested the coding-system independently. Besides IW as first rater, the second rater (OS) was a pharmacist experienced in qualitative studies and not involved in data collection and data analysis. Their results were assessed in a closed-loop process through modifications with subsequent revision and retesting until finalisation. Code-development and coding was performed using MAXQDA (Verbi Gmbh, Berlin, version 12). The complete coding-system is provided in Supplement 2.

For each main code the contents of the associated subcodes were paraphrased. These paraphrases were then summarised for the different professions in PD-group and CG. Next, a synopsis was created for the conclusions from each main code separately for both groups. Supporting aspects for the implementation of MR were extracted from these summaries and phrased as success factors [25]. Conclusions mentioned in the PD-group and CG were rated as not relevant or unique because these strategies were mentioned in both groups but failed in the CG. Finally, the different success factors from the main codes were subsumed and doublings were removed. Hence, a final set of unique or important aspects and strategies to foster MR as a cognitive service in community pharmacies could be developed.

## Results

In the 3A-project 301 certified pharmacies were enrolled, of which 171 (57%) reached an average of x̅ 4.7 [range 1–14] documented MR. Twenty-two pharmacies (eleven per group) were included. Pharmacies in the PD-group conducted a minimum of five MR up to a maximum of 14 MR during the inclusion timeframe. Data saturation was reached after 44 interviews (24 PD-group, 20 CG). Table 1 shows the results of the recruitment process.

**Table 1:** Characteristics of pharmacies and participants

		Positive Deviance-Group	Control-Group
Pharmacies N^a^	Total	8	7
	Small town	2	3
	Medium-size town	4	3
	Large city	2	1
Professional group N^a^	Owner	8	7
	AMTS-MA^b^	8	7
	Technician	8	6
	^a^N $\left( {{{\bar{\text{x}}}}} \right)$ Pharm. staff	${{{\bar{\text{x}}}}}$ 14.5 [5–14]	${{{\bar{\text{x}}}}}$ 11,1 [5–24]
	% $\left( {{{\bar{\text{x}}}}} \right)$ Pharmacists	${{{\bar{\text{x}}}}}$ 33.6% [14%–60%]	${{{\bar{\text{x}}}}}$ 34.4% [18%–50%]
Female, N^a^ (%)		20 (83%)	14 (70%)
Female Professional group N^a^ (%)	Owner	4 (50%)	2 (29%)
	AMTS-MA^b^	8 (100%)	6 (86%)
	Technician	8 (100%)	6 (100%)
Age (years), mean, SD, range	Owner	46.9 ± 8.1 [38–59]	50.0 ± 9.8 [38–65]
	AMTS-MA^b^	39.8 ± 10.6 [28–56]	40.0 ± 12.2 [29–61]
	Technician	35.7 ± 8.4 [29–53]	36.8 ± 14.5 [25–55]
Years in practice, mean, SD, range	Owner	15.9 ± 8.5 [6–32]	23.0 ± 9.9 [11–38]
	AMTS-MA^b^	13.6 ± 10.7 [2–32]	12.0 ± 12.1 [3–35]
	Technician	14.4 ± 7.2 [6–30]	10.8 ± 10.2 [2–27]

*SD* Standard deviation, *Pharm*. Pharmaceutical

^a^N = Number

^b^AMTS-Ma AMTS-manager

Interviews were conducted in German, hence the codings and quotations presented were translated into English with support of a native speaker aiming to reflect the *true* meaning as accurately as possible.

Five main codes emerged from the content analysis representing the important themes:

- Organisational aspects of medication reviews
- Execution of medication reviews
- Collaboration within the medication review process
- Personal attitude towards medication reviews
- Benefit of medication reviews for community pharmacies

### Organisational aspects of medication reviews

This main code was defined as text passages describing organisational structures, prerequisites, and resources relevant for the execution of MR. Thirteen organisational aspects were identified. A complete list of all success factors is displayed in Supplement 3a, quotations to exemplify the development of the different codes are listed in Supplement 4. Involvement of the entire team of the pharmacy deemed most important. Team meetings should include education about the MR-process, roles of the different team-members, patient identification and recruitment, and benefit of MR. Additionally; ongoing education and specialisation of pharmacists in different diseases were emphasised. Participants of the CG did not mention any of these topics.

### Execution of medication reviews

Text passages encoded within this main code referred to professional requirements and support used to conduct MR. Analysis resulted in ten success factors (Supplement 3b). Appropriate identification of eligible patients was rated crucial in the PD-group. Identification criteria were patients with polymedication, patients overwhelmed with their medication, and patients experiencing side-effects.

Most participants used more than one data source to conduct a MR, mainly the patient´s medication list, whereupon the *brown-bag* (patient brings all her/his actually used medication) was considered the most important source of information about the current medication. Usage of clinical-decision-support systems like the ABDA-database, available in all German pharmacies, is common. In addition, pharmacies in the PD-group used external resources like the drug-information-centre of the Pharmacists` Chamber in Westphalia-Lippe. Further important measures identified in the PD-group were information exchange within the entire pharmacy-team on patients receiving MR and giving feedback to technicians about patients identified by them. Documentation plays a pivotal role for implementation of MR. Optimally, specific documentation forms are adapted for the individual pharmacy and incorporated into the software making MR-results visible for all team-members. Further relevant aspects were a need for more standardisation of this service and a change in personal attitude of each team-member towards MR, which must be recognised as meaningful and valuable for the patient and the pharmacy. Conducting MR also requires routine and self-esteem of the particular pharmacist.

### Collaboration within the medication review process

Statements towards collaboration with patients/caregivers and practitioners were allocated to this main code. Practicioners involved in MR conducted in community pharmacies are usually general practitioners however; medical specialists are contacted during the MR process as well. Three sub-codes were developed during analysis revealing eleven success factors: *General aspects* (N = 3), *Collaboration with prescribers* (N = 5) and *Collaboration with patients* (N = 3), (Suppl.3c). Members of the PD-group show their competencies on a daily basis e.g. with interaction- and plausibility-checks which leads to a better visibility of pharmaceutical expertise, resulting in a better cooperation with prescribers, and higher acceptance as health-care professionals by patients and practitioners. Members of the PD-group had a positive attitude towards MR and demonstrated fewer difficulties in offering this service to patients and communicating with prescribers. Intensified collaboration with nursing homes (German pharmacies need a contract to provide medication for nursing homes) by offering MR to residents facilitated the implementation process of MR because nurses can carry out patient identification and information on the complete medication is available in the pharmacy. Both interview groups stated that remuneration through insurance companies, which is only the case in local projects in Germany, would support acceptance.

In general, participants in the PD-group mentioned good collaboration with the practitioners in contrast to members of the CG. Information of practitioners about MR, definition of tasks and establishing of communication-channels were mentioned as necessary before starting this service. Working in rural areas can possibly be an advantage for collaboration with practitioners but data was inconclusive.

The PD-group-members thought themselves as mediator between patient and practitioner whereby members of the CG regarded themselves as competitor or as helpmate of the practitioners reversely*.*

### Personal attitude towards medication reviews

This code summarises statements, which describe how motives and personal engagement influence the implementation of MR. The synopsis of all statements and comparison of PD-group and CG resulted in eleven important factors (Suppl. 3d).

Participants of the PD-group showed a different attitude towards their patients/clients. The ratio of the frequency of the use of the terms *patient* and *customer* was 1.8 in the PD-group compared to 3.5 in the CG. We identified a positive attitude towards MR as the main factor to support implementation of this service. This perception appeared in all three professional groups, and leads to increased professional satisfaction and enhances the attractiveness of the working-place. In both interview-groups, support by the pharmacy-owner/branch-manager was rated as very important whereby participants of the PD-group experienced this in a positive way (MR during working hours, new structures to offer this service, paid continuing education). Participants in the CG missed support or described themselves as *lone warrior* and MR as *stressful.* In the CG technicians showed interest in MR or conducted already small brown-bag-reviews. In contrast, AMTS-managers in the CG felt uncomfortable to take over responsibilities in the context of MR.

### Benefit of medication reviews for community pharmacies

Passages that correlate with benefits of MR are subsumed in this code. Four success factors were identified (Suppl. 3e). The possibility to demonstrate professionalism in healthcare was the main benefit in the PD-group. This aspect was detectable in all professional groups, mainly in the group of pharmacy-owners. Pharmacy-owners and technicians in the CG did not mention this topic at all. Increased customer loyalty was mentioned in the CG as benefit. Some interviewees in the CG, mostly the trained pharmacists, did not see any benefit at all. Economic benefits were coded twice as often in the PD-group achieved through acquisition of new patrons, dispensing of more medication related to increased adherence and using MR as differentiation to online pharmacies. Remuneration of MR itself is not a successfactor. Pharmacies in the CG were eligible for payment in the 3A-project but did not conduct MR at all. Increased attraction of the working-place and motivation of personnel were discussed only in this group.

### Final set of success factors for the implementation of medication reviews

All factors derived from the main codes were summarised and doublings eliminated. A summary of all success factors from each main code is provided in Supplement 5. Based on these results, we compiled a final set of 33 success factors (Table 2). The different factors were grouped in organisational (25) and individual factors (8).

**Table 2:** Final set of success factors for the implementation of medication reviews in community pharmacies

Organisational Factors	
Scope	
Team	Involve the entire team of the pharmacy Integrate technician actively in the MR^a^ process esp. in acquisition of patients Provide feedback to technicians for MR^a^ Document the results visible for the entire team
Training, continuing education	Provide regular training sessions for all team members in process, patient identification and communication, and benefit of MR^a^ Offer chances for ongoing education in pharmacotherapy Acknowledge time for education as working hours
Personnel structure	Restructure workflow within the pharmacy team and allocate tasks according to competencies Incorporate time for MR^a^ into the schedule, provide time as *office-time* Execute MR^a^ during working hours Assure execution of reviews without interruption
Professionalisation of the service	Use standardised material for execution of MR^a^ included in software Equip pharmacy with additional databases and literature Generate a template for written results for patients and prescribers Develop a standard for the organisation of appointments
Patient acquisition	Define patient criteria for acquisition Lable eligible patients in pharmacy software Start providing MR^a^ for patrons and/or nursing home residents
Collaboration	Inform practitioners personally prior to start MR^a^ Define competencies and determine communication channels Discuss first MR^a^ face-to-face with practitioners Organise regular meetings with practitioners
Advertisment	Use social media to communicate the service Utilise branded promotion material Communicate adequate with the different target groups

Individual Factors	
Be ready to overtake responsibility Show a positive attitude towards MR^a^ Demonstrate pharmaceutical competencies in daily routine Specialise in specific topics (disease states/medication classes) Enhance skills in patient communication Execute MR^a^ for training purpose to increase routine Foster self-esteem through routine and increased knowledge Serve as motivator and driving force if you are pharmacy owner or branch manager	

^a^*MR* Medication review

## Discussion

Until now, MR in Germany are not broadly implemented as a pharmaceutical practice service and mainly conducted within projects like the *Apo-AMTS-programme*. Positive Deviance is a unique approach in health service research. A distinctive feature is the focus on *how* things are done instead of *what* is done [12, 13]. This design enabled us to derive factors for an effective implementation of MR in community pharmacies, which are internally generated (bottom-up) from successful participants, the so-called PD-group. Combining the PD-group with an additional CG, which is not a prerequisite for PD-studies, made it possible to *sharpen* the particular results and made it possible to extract a possible formular for success [14]. The included pharmacies were evenly distrubuted across Westphalia-Lippe and did not show relevant discrepancies according to demographics of the interviewees. Pharmacies in the CG had a smaller average number in pharmaceutical staff (${{{\bar{\text{x}}}}}$ 11.1 vs. ${{{\bar{\text{x}}}}}$ 14.5) but a similar percentage of pharmacists (${{{\bar{\text{x}}}}}$ 34.4% vs. ${{{\bar{\text{x}}}}}$ 33.6%). Therefore, these success factors can be considered as feasible within existing resources. Using our findings can lead to new strategies to foster the implementation of MR-services in community pharmacies on a larger scale, and to reevaluate and adjust existing measures and tools. The results strongly emphasise the involvement of the entire pharmacy-team and highlight the role of technicians. This is consistent with previous studies showing that incorporation of technicians improves efficiency of MR and reduces barriers to implementation [1, 26–30]. In addition to the advice *incorporate technicians,* we could add information on how this can be done. For Germany, this means that technicians acquire patients for MR, assess the medical history, support in documentation, prepare interactionchecks and compile medication lists. Training in patient acquisition, adequate communication and education in process and benefits of MR is crucial. Providing feedback about findings during MR and documentation of the results in patients’ health-records, visible for the team, are reasonable procedures as well. Training in all components of MR is a mayor aspect seen in our and in other studies [28, 31–34]. However, as stated in a study by Roberts et al., time for these trainings needs to be considered [34, 35]. A practical approach identified in our project is to acknowledge time for education and training as working hours. The training-sessions in the MR-process, patient- communication and -identification, took place during team-meetings and were offered repeatedly in all interviewed pharmacies in the PD-group.

How a team in a pharmacy performs is always influenced by and directly connected to the organisational structures of the pharmacy. Problems with time, staffing and inefficient workflow were also mentioned in the literature [26, 31, 36–38]. Pharmacies in the PD-group changed the workflow, offered *office hours* to the AMTS-managers and restructured tasks. Execution of MR during free time at home seems to be relevant in Germany only. Nevertheless, due to changes in organisational structures all MR in the PD-group were conducted during working hours resulting in increased motivation to offer this service.

Besides changes in organisation, the particular service MR needs professionalisation itself. Successful implementation is based on integration of standardised material into the pharmacy-software [1, 37, 39–42]. Pharmacies in our study went a step further and made adaptions with their software providers, developed their own material and integrated a validated documentation template developed for Apo-AMTS. Usage of additional decision support systems and literature are well known approaches [35, 43, 44]. In contrast, the implementation of an external service to provide professional support run by the Pharmacists` Chamber in Westphalia-Lippe is unique.

To build a relationship with patients, AMTS-managers used personalised cards for appointments and included these appointments into the pharmacy-calendars. Provision of written results in a branded folder was resonable to demonstrate professionalism. Professionalisation of the service, which includes demonstrating a health professional attitude combined with well-defined organisational structures and comprises a definiton of criteria to identify patients for MR. Polymedication, experience of insecurity or side effects, recent discharge from hospital and living in nursing homes are widely accepted selection criteria also seen in our study. Labeling of eligible patients in the pharmacy’s health-records was identified as useful for the selection process as an additional measure. Overdrawn expectations of patients, a lack of interest and incongruity between pharmacists` and patients` expectation often hamper the acceptance of MR which was also addressed by the interviewees of the CG [35, 37, 45]. Pharmacists from the PD-group overcome this barrier by starting to offer MR to patrons and nursing home residents. Doing so, they gained experience and routine in providing and executing this service. In addition, training in adequate patient-communication during team-meetings is identified as supportive.

Collaboration with practitioners was detected as difficult. Even if an older study from 1997 states that the relationship with practitioners has no influence on the execution of MR most publications and our results indicate that collaboration with GPs who are mainly adressed within the context or MR is still difficult [46]. Pharmacists are seen as *shopkeepers* who sell a service and not as patient-centered healthcare-professional and awareness of the role in community is lacking [47–53]. To optimise collaboration with GPs, it is of utmost importance to know each other in person and to increase awareness of the respective education, the fields of competence, working conditions and/or statutory duties [49, 54]. Strategies to overcome these problems identified in our study were conducting one-on-one interviews with GPs prior to start the service with the aim to reach agreement about competencies, standard procedures and communication channels. We also recommend discussing first MR with practitioners in person to achieve positive experience with MR for both, pharmacist and GP. These approaches led to more acceptance and a consolidated relationship in other studies [49, 55, 56]. To foster relations, regular meetings eased collaboration.

In addition to these strategies, promotion of this service is of importance. This aspect was mentioned by Kotter but did not appear in literature later [45]. We identified useful measures like the use of branded promotion material (flyer, poster, video) and integration of social media like Facebook, Twitter and using the pharmacy`s homepage.

Remunaration of MR is often discussed as necessary for implementation. In Germany, patients usually pay for MR. Only in small projects as the *3A*-*project* pharmacies receive remuneration. Results from our analysis as well as literature are ambiguous [26, 36, 57, 58]. Taking 80€ for one MR into account, which is close to an adequate payment, the low percentage of pharmacies conducting MR within the 3A project (57%) shows that remuneration is not a keyfactor for the implementation. Besides, when this fact is mentioned in both groups here, it should not be considered as a definite factor for success. Nevertheless, striving for remuneration in the future is reasonable.

Optimisation of the organisational factors is a prerequisite for implementation but a positive attitude towards MR, self-esteem of the pharmacists and readiness to take responsibility are crucial factors besides the willingness of pharmacy-owners/branch-managers to support and motivate their employees [35, 58–60]. We can add from our results that demonstration of pharmaceutical competencies in daily routine is the necessary basis to go to the next step and perform MR. Specialisation in particular topics like specific diseases increases self-esteem and confidence of the pharmacists and accelerates the process.

A limitation is that we did not weight these success factors. Therefore, a conclusion cannot be drawn in respect of the quantitative meaning of each of these factors. In addition, it is not possible to assess how these factors are linked together and the relation, respectively the mutual impact of the different factors on each other cannot be judged. This needs to be a matter for further studies. Conducting this study only in one region and with specially trained pharmacists is a possible limitation and needs to be considered if these results are transferred. Nevertheless, we assured by the sampling method that there was no difference between rural areas and larger cities.

## Conclusion

Using the PD-approach combined with a control-group, the results from this study enhance knowledge about strategies to improve implementation rates of MR, the success factors identified are robust, and credible.The promotion of widespread uptake of respective measures might foster the implementation of MR in community pharmacies. Further research is necessary to test the efficacy of these success factors in a larger sample.

## Supplementary Information

Below is the link to the electronic supplementary material.

Supplementary file1 (DOCX 17 KB)

Supplementary file2 (DOCX 14 KB)

Supplementary file3 (DOCX 21 KB)

Supplementary file4 (DOCX 19 KB)

Supplementary file5 (DOCX 19 KB)

## Data Availability

The Standards for Reporting Qualitative Research (SRQR) checklist was used to report this study. The datasets generated and analysed during the current study are available in German from the author on reasonable request.
