# Supplementary material for: Identification of factors for a successful implementation of medication reviews in community pharmacies: Using Positive Deviance in pharmaceutical care
Source: Int J Clin Pharm. 2021 Aug 6;44(1):79–89. doi: 10.1007/s11096-021-01315-1 (PMC8866257; doi:10.1007/s11096-021-01315-1)
Supplement: Supplementary file 1 — Supplementary file1 (DOCX 17 KB) [file 11096_2021_1315_MOESM1_ESM.docx]

Supplement 1: Interview-Guide

**Introduction and briefing**Verification of confidentiality and anonymity
Request for frank statements

Do you have general questions concerning this project?

**Questions for AMTS-Manager**

**Implementation of the service medication review**I would like to hear from you how you implemented the service “medication review” within your pharmacy.

- How do you organize medication review in your pharmacy in general?
- How do you identify patients eligible for a medication review?
  - What is your approach, what is supportiv?
  - What do you regard as barrier for identification of patients?
- What material do you use in your pharmacy to address or inform patients about your service?
- How do you proceed to address patients in practice?
- What processess were changed or newly implemented to offer medication reviews?
  - Can you describe problems you see in this context?
- What are the benefits for your pharmacy conducting medication reviews?

**Execution of medication reviews**I am interested in how you perform a medication review

- How do you manage appointments? What material do you use e.g. reminder?
- On which days do you offer medication reviews?
- What areas or rooms do you have available in the pharmacy?
- How much time do you generally need for the first appointment?
- How much time do you need for the AMTS-examination?
- At what time and where do you conduct the AMTS-examination?
- Please describe the data-bases and resources you use!
- Is a QM-process available?
- How much time does the owner/branch manager provides you to conduct a medication review?
- What processess were changed or newly implemented to conduct medication reviews?
  - Can you describe problems you see in this context?
- What trainings did you provide for the technicians?
- Can you give a description of your documentation template?
- How did you organize to collaborate with the physicians?
- What is the remuneration for the medication reviews?

**Personal attitude**What do you think is your personal attitude to conduct medication reviews?

- Can you tell me something about your motivation to offer medication reviews to patients?
- How do you handle denial of this offer?
- Can you list barriers you had to resolve?
- What is your personal benefit when you conduct medication reviews?
- How important is support of the pharmacy owner/brand manager for you?
- Is there any further support you would like to see for further successful provision of the service medication review?

**Questions for technicians**

**Implementation of the service medication review:**I would like to hear from you how the service “medication review” is implemented within your pharmacy.

- Can you describe how you are involved in this service?
- What specific information did you receive in conjunction with medication reviews?
- How do you identify patients eligible for medication reviews in your daily routine?
  - Please describe how you proceed!
  - Can you list some obstacles you experience with approaching the patients?
- What material do you use in your pharmacy to address or inform patients about this service?
- How do you proceed to address patients in practice?
- What processess were changed or newly implemented to offer medication reviews?
  - Can you describe problems you see in this context?
- What are the benefits for your pharmacy conduction medication reviews?
- What kind of feedback do you get from the AMTS-manager who conducted a medication review with a patient you identified?
- How reasonable is it to you to be involved in this service?

**Execution of medication reviews**I am interested to hear how medication reviews are conducted in your pharmacy practically.

- How do you make appoinments with patients for medication reviews?
- What days are choosen to make appointments?
- Where are patient interviews executed in the pharmacy?
- Please describe the different tasks you perform within the scope of medication reviews!
  - What training did you percive for that?

**Personal attitude**I would like to know, what is your personal interest to offer medication reviews to patients?

- What is your motivation to offer medication reviews?
- How do you react if patients reject that offer?
- Can you describe some barriers you had to overcome?
- What are your personal benefits to be involved in medication reviews?
- Is there any further support you need or you would like to have to offer this service more successful?

**Quotations for pharmacy owners / branch managers**

**Implementation of the service medication review:**I would like to hear from you how the service “medication review” is implemented within your pharmacy.

- Please describe how you implemented medication reviews in your pharmacy!
- Did you change routines or what kind of new processes did you implement?
  - What problemes did appear within the context of the implementation of this service?
- What different material did you use?
- How do you motivate your staff to offer and conduct medication reviews on a regular basis?
- What are the benefits for your pharmacy conducting medication reviews?

**Execution of medication reviews**I am interested to hear how medication reviews are conducted in your pharmacy in practice.

- On which days do you offer medication reviews?
- What accomodations are available in the pharmacy?
- What timeframe do you set for the conduction of medication reviews?
- Can you describe how you organized the coopertion with the practitioners?
- What is the remuneration for the medication reviews?

**Personal attitude**I would like to know, what is your personal interest to implement medication reviews?

- What is your motivation to offer medication reviews in your pharmacy?
- What barriers did you have to overcome?
- Please describe your personal benefits why you implemented medication reviews in your pharmacy!
- Is there any further support you would like to see for further successful provision of the service medication review?
