# Supplementary material for: Identification of factors for a successful implementation of medication reviews in community pharmacies: Using Positive Deviance in pharmaceutical care
Source: Int J Clin Pharm. 2021 Aug 6;44(1):79–89. doi: 10.1007/s11096-021-01315-1 (PMC8866257; doi:10.1007/s11096-021-01315-1)
Supplement: Supplementary file 2 — Supplementary file2 (DOCX 14 KB) [file 11096_2021_1315_MOESM2_ESM.docx]

**Supplement 2**: Final coding system with main- and subcodes

| Main Code | Subcode | Sub-Subcode | Sub-Sub-Subcode |
| --- | --- | --- | --- |
| Organisation of MR | Addressing patients | Advertising | Advertising material successful/ not successful/not used |
|  |  | Team education | |
|  |  | Appointments | |
|  | Accommodation |  |  |
|  | Resources | Personnel resources | Sufficient employees/  lack of employees |
|  |  |  | MR during working hours/ MR at home |
|  |  | Knowledge resources | |
|  |  | Technical resources | |
|  |  | Financial resources | |
|  |  | Quality management | Yes/No |
| Execution of MR | Identification of patients | | |
|  | Patient data assessment | Resources of patient data | |
|  | Medication reconciliation | Performance | |
|  |  | Documentation | |
|  |  | Duration | Duration appropriately/  too long |
| Collaboration within the MR process | Prescriber | Facilitators | |
|  |  | Barriers | |
|  | Patient | Facilitators | |
|  |  | Barriers | |
| Personal attitude | Pharmacy-owners/branch- managers | Positive statements | |
|  |  | Negative statements | |
|  | *AMTS-managers* | Positive statements | |
|  |  | Negative statements | |
|  | Technicians | Positive statements | |
|  |  | Negative statements | |
| Benefit of MR |  | | |

MR=Medication Review
