# Supplementary material for: Identification of factors for a successful implementation of medication reviews in community pharmacies: Using Positive Deviance in pharmaceutical care
Source: Int J Clin Pharm. 2021 Aug 6;44(1):79–89. doi: 10.1007/s11096-021-01315-1 (PMC8866257; doi:10.1007/s11096-021-01315-1)
Supplement: Supplementary file 3 — Supplementary file3 (DOCX 21 KB) [file 11096_2021_1315_MOESM3_ESM.docx]

**Supplement 3a-e**

**3a** Successfactors derived from the main-code “**Organisation of medication reviews**”

| Scope | Successfactors |
| --- | --- |
| Addressing patients | - Advertisement with pharmacy individualised material in a broad range of media (e.g., social media, poster, newspaper articles…) - Flyer use only for individual patients - Labeling of patients in software-program - Reminder buttons to offer MR at the cash points - Appointments in written form for patients accompanied with contact data of the patient and the responsible AMTS-manager - Repeated educational sessions for the entire team about the MR- program - Training for patient communication relating to MR for pharmacists and technicians |
| Accommodation | - Space/room for patient counselling is equipped as appropriate |
| Resources | - Time for patient interviews and reviews are coordinated with staff availability - Working processes are restructured according to responsibilities of the different profession groups - The review process takes places in the pharmacy with sufficient time - Ongoing education for all professions is mandatory - Specialization in different diseases and/or drug classes within the team |

MR=Medication review

**3b** Successfactors derived from the main-code “**Execution of medication reviews**”

| Scope | Sucessfactors |
| --- | --- |
| Identification of patients | - Determination of identification criteria for patients eligible for a MR - Training sessions for patient identification - Patrons are a convenient target group - Adequate communication strategies to identify patients |
| Patient data assessment | - Important data sources are:   - Brown-Bag   - Medication plan   - Customers` files |
| Medication reconciliation | - Pharmacies need to be provided with adequate databases and literature - Material to conduct the medication reconciliation needs to be standardized and optimally integrated into the pharmacy software - Medication reconciliation should be performed without interruption - Continuing education for pharmacists and technician in the area of MR and medication therapy safety is crucial - Routine is a key-factor for the implementation of MR   - Directly influences time required and therefore staffing   - Achievement of self-esteem of pharmacists and certainty   - Foundation for readiness to assume responsibility   - MR as regular service instead of a special service |

MR=Medication review

**3c** Successfactors derived from the main-code “**Collaboration with the medication review process**”

| Scope | Successfactors |
| --- | --- |
| General aspects | - Interaction checks and communication of the results with the prescriber/patient in daily routine - Collaboration with nursing homes as entry into MR service - Positive attitude towards MR |
| Prescriber level | - Prescriber information previously to start of MR services - Clarification of task and competencies - Determination of communication channels - Pharmacists/Prescribers meetings on a regular basis - Discussion of first MR always face-to-face |
| Patient level | - Starting MR with patrons to begin with - Presentation of healthcare competencies in daily routine - Custom-tailored advertising and information material |

MR=Medication review

**3d** Successfactors derived from the code “**Personal attitude towards medication reviews**”

| Main-Code | Sucessfactor |
| --- | --- |
| Personal attitude towards MR | - A positive personal attitude towards MRs is essential for conduction of MR - Willingness to overtake responsibility is a prerequisite - Implementation needs a positive attitude and active support of the pharmacy owner - Incorporation of the entire team is essential - Technicians are actively integrated in the MR process - Active feedback is provided to technicians according to patients recruited by them - The pharmacy team receives training in benefits of MRs - Preparedness to handle pharmacotherapeutic questions is a necessity - Continuing education in general and for MRs are part of working hours - Training and routine improve customer-friendly communication - Clear differentiation of the definitions of MR and counselling according to regulatory requirements |

MR=Medication review

**3e** Successfactors derived from the code “**Benefits of medication reviews**”

| Main-Code | Successfactor |
| --- | --- |
| Benefit of MR | - Demonstration of health care related competency in daily routine is the basis for implementation of MR - Improvement of counseling skills of the entire team due to experience form MR fosters the demonstration of health-related competency and lead to more acceptance and increase patient safety in general - Motivation of the staff improves implementation and vice versa - Positive economic aspects convince pharmacy owners to implement MR |

MR=Medication review
