# Supplementary material for: Identification of factors for a successful implementation of medication reviews in community pharmacies: Using Positive Deviance in pharmaceutical care
Source: Int J Clin Pharm. 2021 Aug 6;44(1):79–89. doi: 10.1007/s11096-021-01315-1 (PMC8866257; doi:10.1007/s11096-021-01315-1)
Supplement: Supplementary file 4 — Supplementary file4 (DOCX 19 KB) [file 11096_2021_1315_MOESM4_ESM.docx]

**Supplement 4**
Main codes developed during the qualitative analysis and associated quotations

| **Main code** | **Successfactor** | **Quotations** |
| --- | --- | --- |
| Organisational factors of medication reviews | Inclusion of the team | *“We did inform all colleagues during a team-meeting about medication reviews as our new cognitive service and that this is a new aim for us. [...] There is an ongoing reporting about the progress we made.” [B7.1_04]* |
|  | Teaching of the team Patient recruitment | *“We make sure that they (technicians) know that we offer that service. We had in advance a, together, such a team meeting about that topic. I did explain what it is and they now know how it works so to say. And always, if they realize that something does not run smoothly or he (patient) does not get along or he has crazy many drugs and they see 1000 interaction warnings, then they contact me.” [B1.2_14]* |
|  | Internal communication  Increase of knowledge/Resources | *“We said for example, because we are three pharmacists, we will divide the different domains. That one will work on psychotropic drugs and find information faster, another one the antihypertensive drugs and the third one works a little more on the marginal themes. Simply to increase knowledge and then to communicate internally a little more.” [B2.1_11]* |
| Execution of medication reviews | Patient identification | *“[…] if we realize someone does not get well along with his medication or is discharged from hospital and is just somehow in a changing phase, does actually not know what just happened to him, then we address these problems directly […].” [B1.2_04]* |
|  | Patient data assessment | *“Therefore the brown-bag – method is the best method in my opinion. So, if you write it down just out of your mind that is bad.” [B4.2_01]* |
|  | Communication within the team/ Feedback | *“That is a fact that this happens in our pharmacy. We, I try to get the information what happened to the patient, what did my colleagues detect.”*  *[B2.3_12] “So you will get a feedback?”[I] “Yes, that`s what we get. That is very important.” [B2.3_12]* |
|  | Documentation | *“[…] we try to make it apparent, in the computer, so everyone can see what was done. That you can say with a quiet conscience that was all done. Exactly.” [B5.3_22]* |
|  | Organisation of workflow | *“Because it is an enormous expenditure of time. You can`t do it just quickly during the normal work.” [C.1_11]* |
| Cooperation within the medication review process | Cooperation with practitioner | *“And then it happens that doctors send their patients to us to discuss the medication and to give a feedback.”  [B1.2_14]* |
|  | Cooperation with practitioner | *“When we started the project, we had the possibility to talk about the first patients in the doctor`s office for half an hour. We explained the project and what kind of support we can offer and this really worked out very well.” [B3.2_19]* |
|  | Cooperation with patients | *“There is always the question if they can reduce their number of drugs: can you check this out please, my doctor has no time for that or my doctor, she doesn`t want to do that. […].” [B1.1_15]* |
|  | Cooperation with patients | *“Ok, I believe that patients are much more open minded, they have less constrains to say something compared to the doctor’s office. […] in the pharmacy these questions about these things are asked much more often.” [B5.1_16]* |
|  | Cooperation with patients | *“[…] because customers carry it out into the world that we offer something like that, that we do a good job and that they benefit respectively.”  [B3.1_17]* |
|  | Cooperation with patients | *“I just believe if patients have not to pay this service on their own and it would be a service, like I see my doctor with my insurance card nothing would hinder implementation.” [ B6.2_05]* |
| Personal attitude towards medication reviews | Positive attitude -  employed pharmacist | *“Because it is a challenging task and because I can contribute my medicinal and pharmaceutical knowledge. I believe everybody likes to do this because it makes more sense. I think it brings a new meaning to the profession.” [B4.2_01]* |
|  | Positive attitude -  pharmacy owner | *“[…] especially my number of pharmacists, there I am very good equipped because I just want to work with competent staff.” [B6.1_10:]* |
|  | Positive attitude -  employed pharmacist | *“It is a matter of time or if you always have something else to do in between then it is more stressful as if you see something; that you have positive results, must I say.” [BG.2_20]* |
| Benefits of medication reviews for community pharmacies | Perception as healthcare professional | *“Perception of our profession changes. Especially patients who experienced that (MR) and that they return and ask more detailed questions and they say that they get better explanations than from their doctor and that we simply have more competencies in medication.” [B3.2_19]* |
|  | Staff motivation | *“For my staff it is in fact nice because I personally believe it is a motivation for the staff because it is the essence of the work of a pharmacist to do something like this.” [B3.1_17]* |
