# Supplementary material for: Identification of factors for a successful implementation of medication reviews in community pharmacies: Using Positive Deviance in pharmaceutical care
Source: Int J Clin Pharm. 2021 Aug 6;44(1):79–89. doi: 10.1007/s11096-021-01315-1 (PMC8866257; doi:10.1007/s11096-021-01315-1)
Supplement: Supplementary file 5 — Supplementary file5 (DOCX 19 KB) [file 11096_2021_1315_MOESM5_ESM.docx]

**Supplement 5**

Entity of all successfactors derived from the different main codes

| Scope | Main Code |
| --- | --- |
| **Important factor for implementation on organisational level** | |
| Addressing patients | - Advertisement with pharmacy individualised material in a broad range of media (e.g. social media, poster, newspaper articles…) - Flyer use only for individual patients - Labeling of patients in software-programme - Reminder buttons to offer MR at the cash points - Appointments in written form for patients accompanied with contact data of the patient and the responsible AMTS-manager - Repeated education sessions for the entire team about the MR- programme - Training for patient communication relating to MR for pharmacists and technicians |
| Accommodation | - Space for patient counselling is equipped as appropriate |
| Resources | - Time for patient interviews and reviews are coordinate with staff availability - Working processes are restructured according to responsibilities of the different profession groups - The review process takes places in the pharmacy with sufficient time - Ongoing education for all professions is mandatory - Specialisation in different diseases and/or drug classes within the team |
| **Important factor for implementation on execution level** | |
| Identification of patients | - Determination of identification criteria for patients eligible for a MR - Training sessions for patient identification - Patrons are a convenient target group - Adequate communication strategies to identify patients |
| Patient data assessment | - Important data sources are:   - Brown-Bag   - Medication plan   - Customers` files |
| Medication reconciliation | - Pharmacies need to be provided with adequate databases and literature - Material to conduct the medication reconciliation needs to be standardised and optimally integrated into the pharmacy software - Medication reconciliation should be performed without interruption - Continuing education for pharmacists and technician in the area of MR and medication therapy safety is crucial - Routine is a key-factor for the implementation of MR   - Directly influences time required and therefore staffing   - Achievement of self-esteem and certainty   - Foundation for readiness to assume responsibility   - MR as daily routine instead of a special service |
| **Important factor for implementation on cooperation level** | |
| General aspects | - Interaction checks and communication of the results with the prescriber/patient in daily routine - Cooperation with nursing homes as entry into MR - Positive attitude towards MR |
| Prescriber level | - Prescriber information previous to start of MR services - Clarification of task and competencies - Determination of communication channels - Pharmacists/Prescribers meetings on a regular basis - Discussion of first MR always face-to-face |
| Patient level | - Starting MR with patrons to begin with - Presentation of healthcare competencies in daily routine - Custom-tailored advertising and information material |
| **Important factor for implementation on personal level** | |
| Personal attitude | - A positive personal attitude towards MRs is essential for conduction of MR - Willingness to overtake responsibility is a prerequisite - Implementation needs a positive attitude and active support of the pharmacy owner - Incorporation of the entire team is essential - The pharmacy team receives training in benefits of MR - Preparedness to handle pharmacotherapeutic questions is a necessity - Continuing education in general and for MR are part of working hours - Technicians are actively integrated in the MR process - Active feedback is provided to technicians according to patients recruited by them - Training and routine improve customer-friendly communication - Clear differentiation of the definitions of MR and counselling according to regulatory requirements |
| **Important factor for implementation according to benefits of MR** | |
| Benefit | - Demonstration of health care related competency in daily routine is the basis for implementation of MR - Improvement of counseling skills of the entire team due to experience form MR fosters the demonstration of health-related competency and lead to more acceptance and increase patient safety in general - Motivation of the staff improves implementation and vice versa - Positive economic aspects convince pharmacy owners to implement MR |

MR=Medication review
